# Complete Genome Sequence of a Class I Avian Orthoavulavirus 1 Isolated from Commercial Ostriches

**DOI:** 10.1128/MRA.00543-19

**Published:** 2019-07-25

**Authors:** Celia Abolnik, Christine Strydom

**Affiliations:** a Department of Production Animal Studies, Faculty of Veterinary Science, University of Pretoria, Onderstepoort, South Africa; b Deltamune (Pty) Ltd., Centurion, South Africa; DOE Joint Genome Institute

## Abstract

A hemagglutinating virus isolated during routine surveillance in ostriches was sequenced, identified as avian orthoavulavirus 1 (AOaV-1), and classified as a class I genotype 1.2 virus, with recent common ancestors in Eurasian wild ducks. This is the first class I AOaV-1 isolate from Africa and the first identified in ostriches.

## ANNOUNCEMENT

Avian orthoavulaviruses (AOaV; formerly avian paramyxoviruses) are enveloped single-stranded negative-sense RNA viruses found in a diverse range of wild birds, particularly those associated with water. Of the 20 collective avian *ortho*-, *meta*-, and *para*-avulavirus members described to date, AOaV-1 is the most significant from an animal heath perspective, as it contains virulent viruses that cause Newcastle disease in susceptible species. AOaV-1 is further divided into classes I and II, each containing defined genotypes and subgenotypes. Avirulent as well as the virulent genotypes that cause Newcastle disease are classified within class II, whereas class I contains only avirulent viruses and is commonly found in waterfowl and shorebirds (1).

The semiarid Karoo region at the southernmost tip of the African continent is the hub of global commercial ostrich (Struthio camelus) production. These large flightless birds are farmed for meat, leather, and feathers in a free-range production system that brings them into close contact with wild water birds drawn to feeding stations, water sources, and irrigated pastures in an otherwise water-scarce environment. Fecal-oral transmission of viruses maintained in the wild avian reservoir is unavoidable and in the past has been the source of avian influenza virus outbreaks in ostriches (2). In October 2014, tracheal swabs were collected from 3- to 4-month-old commercial ostriches in the Oudtshoorn district for routine surveillance for influenza virus. A hemagglutinating virus was isolated from tracheal swabs in 9- to 11-day-old embryonated specific-pathogen-free chicken eggs through the standard method of inoculation into the allantoic cavity (3).

**FIG 1:**
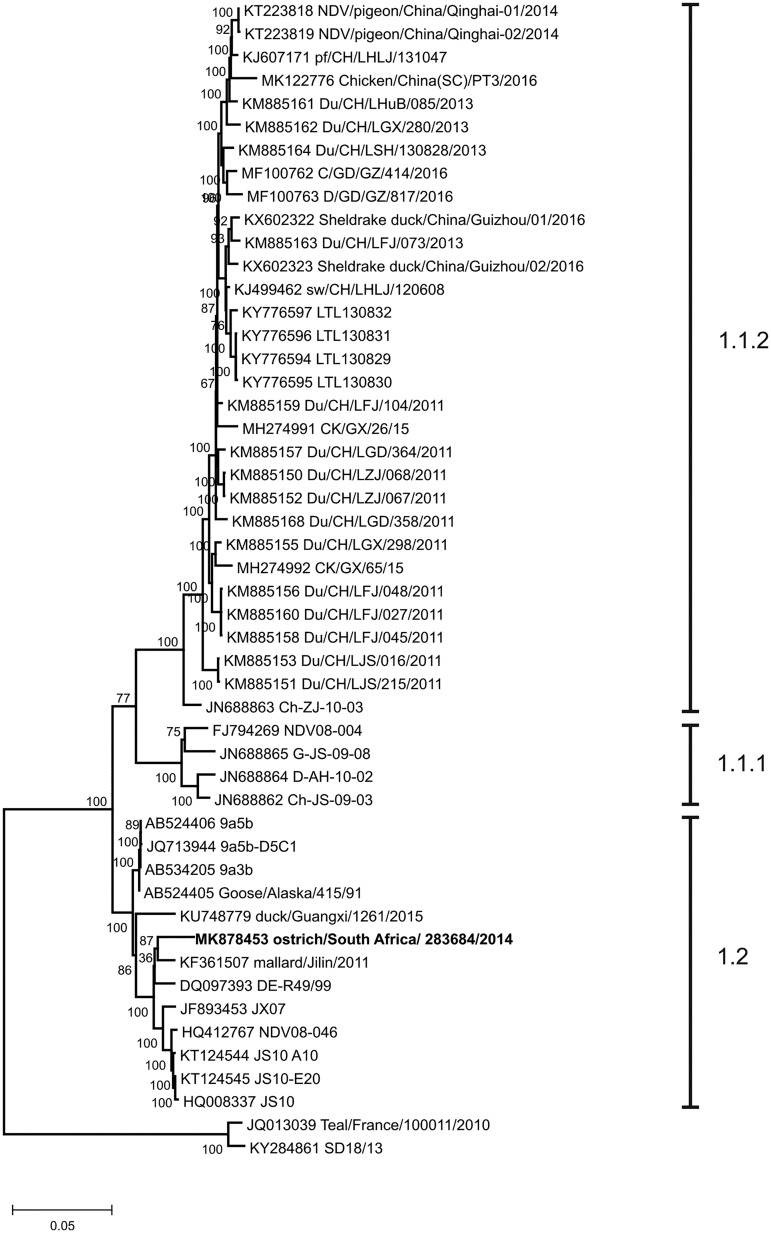
Phylogenetic tree of the fusion protein genes of class I orthoavulavirus 1 strains. Multiple-sequence alignments were prepared in BioEdit v7.5.2 with selected references retrieved from GenBank (accession numbers are to the left of the strain names). Phylogeny was reconstructed using the maximum likelihood statistical method in MEGA v5.5.2, with 1,000 bootstrap replicates. The Tamura-Nei nucleotide substitution model was used, specifying a uniform rate among sites. The tree is rooted with two class I viruses that have not yet been assigned a genotype.

To identify the virus isolate, RNA was extracted with TRIzol reagent (Life Technologies), and adapter-ligated cDNA libraries were prepared with Ion Total RNA sequencing (RNA-seq) v2 and Ion Xpress RNA-seq barcode kits (Thermo Fisher Scientific, Waltham, MA, USA). Massively parallel sequencing was performed on the Ion Proton system (4). In CLC Genomics Workbench v5.1.5, *de novo* assembly (default parameters) of 2,266,530 sequencing reads with a mean length of 71 bp produced a contig of 15,205 nucleotides, along with smaller nonspecific contigs of <1,920 bp. BLAST analysis (https://blast.ncbi.nlm.nih.gov/Blast.cgi) identified the contig as a complete genome of AOaV-1, with 100% coverage and 97.37% nucleotide sequence identity with the closest relative, isolate mallard/Jilin/2011 (GenBank accession number KF361507). Maximum likelihood phylogenetic analysis of the fusion protein genes of ostrich/South Africa/283684/2014 and references retrieved from GenBank indicated recent common ancestry with isolates from wild ducks in Finland, Germany, Russia, Kazakhstan, China, and Japan that are classified as genotype 1.2 within class I of AOaV-1 (Fig. 1) (1). The fusion protein cleavage site motif, a critical determinant of virulence (5), was ^112^GERQERL^117^, i.e., lacking dibasic amino acid pairs and phenylalanine at position 117, thus confirming the avirulent pathotype.

This is the first complete genome sequence for a class I AOaV-1 virus from the African continent and the first from ostriches. It reiterates that South Africa is at the end of a migratory funnel for wild waterfowl that breed in Eurasia and harbor viruses that they are capable of spreading over vast distances.

### Data availability.

The complete genome sequence and associated data of ostrich/South Africa/283684/2014 have been deposited in GenBank under accession number MK878453, BioProject accession number PRJNA549564, experiment number SRX6092860, and BioSample accession number SAMN12091794.
